# A Comprehensive Overview of Network Slicing for Improving the Energy Efficiency of Fifth-Generation Networks

**DOI:** 10.3390/s24103242

**Published:** 2024-05-20

**Authors:** Josip Lorincz, Amar Kukuruzović, Zoran Blažević

**Affiliations:** Faculty of Electrical Engineering, Mechanical Engineering and Naval Architecture (FESB), University of Split, 21000 Split, Croatia; amarkukuruzovic@gmail.com (A.K.);

**Keywords:** 5G, network slice, energy efficiency, software define network (SDN), virtual network function (VNF), third-generation partnership project (3GPP), MNO

## Abstract

The introduction of fifth-generation (5G) mobile networks leads to an increase in energy consumption and higher operational costs for mobile network operators (MNOs). Consequently, the optimization of 5G networks’ energy efficiency is crucial, both in terms of reducing MNO costs and in terms of the negative environmental impact. However, many aspects of the 5G mobile network technology itself have been standardized, including the 5G network slicing concept. This enables the creation of multiple independent logical 5G networks within the same physical infrastructure. Since the only necessary resources in 5G networks need to be used for the realization of a specific 5G network slice, the question of whether the implementation of 5G network slicing can contribute to the improvement of 5G and future sixth-generation networks’ energy efficiency arises. To tackle this question, this review paper analyzes 5G network slicing and the energy demand of different network slicing use cases and mobile virtual network operator realizations based on network slicing. The paper also overviews standardized key performance indicators for the assessment of 5G network slices’ energy efficiency and discusses energy efficiency in 5G network slicing lifecycle management. In particular, to show how efficient network slicing can optimize the energy consumption of 5G networks, versatile 5G network slicing use case scenarios, approaches, and resource allocation concepts in the space, time, and frequency domains have been discussed, including artificial intelligence-based implementations of network slicing. The results of the comprehensive discussion indicate that the different implementations and approaches to network slicing pave the way for possible further reductions in 5G MNO energy costs and carbon dioxide emissions in the future.

## 1. Introduction

Fifth-generation (5G) mobile networks bring new and revolutionary services to users, significantly higher data transfer speeds, and the interconnection of various network devices that will transform the digital world. Considering the amount and price of the resources needed for the complete realization of standalone 5G networks, this advanced technology also brings challenges that need to be addressed, and one of these challenges is certainly related to the increased energy consumption of the mobile network. This is a consequence of the main characteristic of 5G networks, which is related to the need for an increase in the density of base station (BS) installations and higher BS capacities due to the need for the transmission of data at high speeds using broadband channels. Due to these facts, an inevitable increase in 5G network energy consumption occurs. This increased energy consumption has several negative consequences. The first one is reflected in the negative environmental aspects related to increased CO_2_ emissions. According to the available data, the telecommunications industry accounts for approximately 2% of the total global CO_2_ emissions [1]. Additionally, the negative aspect of increased energy consumption is reflected in the increased operational costs of mobile operators, which, in many cases, lead to increased telecommunication service prices. By 2030, in the best-case scenario, it is projected that 8% of the global demand for electrical energy will come from the information and communication technology (ICT) sector [1].

The aforementioned factors indicate that striving towards better energy efficiency (EE) in mobile networks is one of the key goals in the planning, implementation, and operation of 5G mobile networks. Knowing this, EE improvement entails optimizing resources that can lower the energy consumption, mitigate the emission of greenhouse gases, and incorporate renewable energy sources in the power supply of network elements [2]. It is expected that with the full practical global implementation of 5G networks by 2030, the data transfer speed will be increased by a factor of 1000, while, simultaneously, network energy consumption will be reduced by a factor of 2. To achieve such energy efficiency improvements that are 2000 times higher per transferred bit in comparison with the energy efficiency of previous network generations, different solutions need to be implemented in order to enable the optimization of 5G network energy consumption [3]. One of the prominent solutions dedicated to the optimization of 5G network resource usage is a technique known as network slicing. Fifth-generation network slicing enables the creation and existence of multiple independent logical 5G networks within the same physical infrastructure. This offers great flexibility for specific mobile network implementations which can contribute to the reduction in service implementation costs and extension of service offerings, and can ensure better exploitation of the mobile network operator (MNO) resources. In this context, the question that arises is whether the implementation of network slicing in 5G networks can contribute to the improvement in network energy efficiency. Thus, this review paper aims to provide an answer to this question and focuses on analyses of improvements in the energy efficiency of 5G mobile networks that were achieved by employing the technique of network slicing.

### 1.1. Introduction to 5G Network Slicing

The architecture of 5G mobile networks enables the creation and existence of multiple independent logical networks known as network slices (NSs) on the shared 5G networks’ physical infrastructure. Each independent 5G network segment (NS) has its own resource requirements, with the aim of meeting the quality of service (QoS) requirements of each application, user, or specific market. Within the concept of network slicing, each distinct NS, constituting a virtual network, is a self-contained assembly of network resources and functions. These NSs operate autonomously, spanning from initiation to completion, and possess diverse requirements that include energy consumption considerations. Additionally, they feature independent management and control mechanisms that are customized to align with the precise requirements of an operator, application, service, user, or a specific vertical market [4].

**Figure 1:**
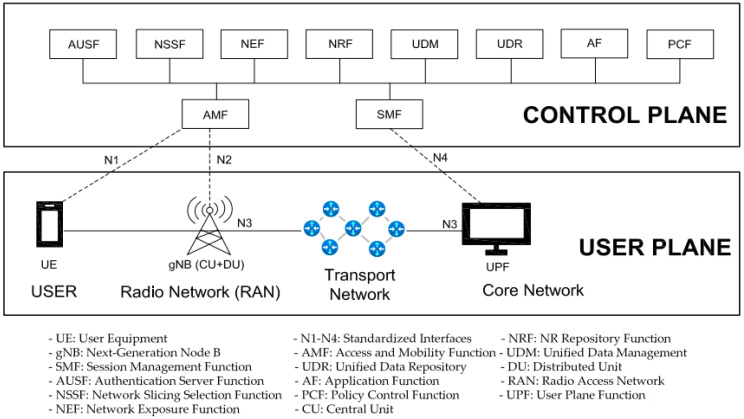
5G standalone network [5].

In Figure 1, a standalone (SA) 5G network is shown. This implies that in practical network realization, there are also non-standalone 5G mobile network deployments that rely on the integration of the established fourth-generation (4G) long-term evolution (LTE) network alongside a 5G radio access network (RAN). In contrast, SA 5G networks operate independently by utilizing both a 5G RAN and a native cloud 5G core. This is the main reason why non-standalone 5G networks do not have network slicing capabilities and they are beyond the scope of this paper.

From Figure 1, it can be seen that the 5G network is composed of a user plane consisting of the user equipment (UE), RAN, transport network (TN), and user plane function (UPF) of the core network (CN). Thus, the user plane encompasses data flow, data encapsulation, and particularities related to data transmission. On the other hand, the control plane comprises the transfer of control and management information and takes into account all functionalities unrelated to the actual communication of user content. The control plane manages session administration, verifies access rights for services or NSs, distributes and enforces service rules, and stores user data. Network slicing can be applied to some of these components, establishing distinct entities for each NS, or it can allow for a single entity to be shared across multiple NSs. Typically, in practical realizations of 5G network slicing, the access and mobility function (AMF) is shared among several NSs, whereas the session management function (SMF) and UPF are generally allocated to each NS separately. The rationale behind distributing the AMF among slices is to minimize signaling from the UEs when the utilized services span across various NSs. Alongside the AMF, the unified data management (UDM) and network slicing selection function (NSSF) are shared among multiple NSs, encompassing all NSs to simplify NS management complexity.

**Figure 2:**
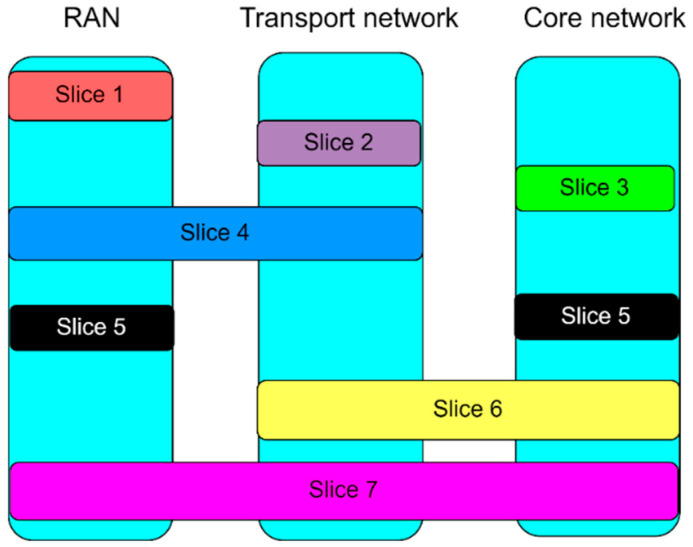
Network slicing in 5G.

Although TN is typically owned by the MNO, in practice, different allocations of TN infrastructure elements in 5G network slicing can be seen. According to Figure 2, network slicing can be applied separately to each architecture element of the 5G mobile network or it can be realized as a combination of some parts or all elements of the 5G standalone network. If an NS encompasses the CN part, the TN part, and the RAN part of the mobile network (as presented for slice 7 in Figure 2), it is referred to as end-to-end (E2E) network slicing.

### 1.2. Overview of Main Contributions

Concerning network EE, implementing any type of network slicing other than E2E NS is not an overall solution for satisfying the ambitious goals dedicated to improving the EE of 5G networks. It can rather be treated as an improvisation in a specific use case or a transitional solution until the establishment of E2E NS in 5G networks. Examples of such slicing concepts are presented in Figure 2 as slice 1 (RAN slicing), slice 2 (TN slicing), slice 3 (CN slicing), etc. The increased cost of energy and ecological awareness has led to the development of the Energy-Aware Virtual Network Embedding (EA-VNE) concept [6,7,8] in which the goal is to optimize the network’s energy consumption through the allocation of virtual network demands to physical network resources. Since the implementation of E2E network slicing and EA-VNE involves establishing numerous virtual networks on a shared 5G network physical infrastructure, technologies such as network function virtualization (NFV) and software-defined networking (SDN) become key enablers of 5G network slicing [9]. Apart from these two technologies, important enablers of network slicing include edge and cloud computing [10]. All of these concepts will be analyzed in the rest of the paper as means for improving 5G network energy efficiency through the implementation of network slicing.

Thus, the contribution of this paper is to highlight the advantages of employing network slicing as a means of enhancing the EE of 5G networks. Based on a review of the published literature, it will be shown that the topic of improving 5G network EE through network slicing has not been thoroughly addressed at the time of writing this paper, and this paper aims to fill this gap. Key performance indicators (KPI) for determining the EE of 5G network slices will be presented, along with research related to standardization led by the third-generation partnership project (3GPP) in the field of defining the energy efficiency of NSs. The paper also discusses energy efficiency in 5G network slicing lifecycle management. To show how efficient network slicing can optimize the energy consumption of 5G networks, versatile 5G network slicing use case scenarios, approaches, and resource allocation concepts in the space, time, and frequency domains are discussed, including artificial intelligence-based implementations of network slicing. The paper also discusses ideas for further research in the field of improving 5G network EE through the implementation of network slicing.

The rest of the paper is organized as follows. Section 2 overviews scientific papers published on the topic of the impact of network slicing on 5G network EE. Section 3 deals with a detailed overview of network slicing and standardized types of NS use cases. Section 4 discusses elements of 3GPP standardization dedicated to the implementation of NS on network EE. Section 5 provides a general description of the impact of network slicing on 5G network EE, as well as ideas for possible further research on improving the EE of 5G networks through the implementation of NS. Section 6 concludes the paper.

## 2. Related Work

Since NFV and SDN are base technologies for establishing NSs, each of these technologies can be used for exploiting specific methods and approaches that can contribute to network energy savings improvements. The fundamental idea of SDN networks relies on a tangible division between the control and data plane of SDN networks. According to [11], the SDN concept can offer solutions for improving network E, including the energy-efficient plane and the data plane (Figure 1). This is in contrast with conventional networks, where each networking device incorporates both the data and the control planes. In the case of sliced 5G networks realized using NFV, the control plane can manage the data plane of multiple networking devices from a centralized location (Figure 1). This means of network realization sets the ground for a possible improvement in 5G network EE and energy savings, since networking devices only use power to perform those tasks that are directed by the control plane. Table 1 provides an overview of the main areas of research, which are based on the implementation of network slicing in 5G networks to improve 5G networks’ energy efficiency.

Solutions for improving network energy efficiency, realized through the implementation of SDN, can be classified into three models that are based on traffic-aware effects, end-host-aware effects, and rule placement effects (Table 1). The traffic-aware model implies that the SDN controller periodically updates the status of the router and switch states in the transmission network, and this information is used to optimize energy consumption through the decision to turn off certain devices in conditions of reduced traffic. The goal of the end-host-aware model is to find routes with a number of network nodes and connections linked to them, creating conditions for the implementation of the energy-efficient traffic-aware model. In this model, the SDN controller groups multiple connections over the same network nodes and links and thereby creates some network elements (NEs) that can be in idle (power-saving) mode. This contributes to a significant reduction in consumed energy, while ensuring quality of service (QoS). Considering that SDN switches use ternary content addressable memory (TCAM) to store the forwarding rules obtained from the SDN controller, and the access to TCAM directly impacts the increased energy consumption, the rule placement model attempts to reduce the number of TCAM entries by using wildcard entries or default routes that the SDN switch will follow. Since SDN is one of the key technologies for the realization of NSs, i.e., the establishment of NS is not possible without the use of SDN, this implies that these three models open space in the network slicing concept for significant energy consumption savings that can potentially be achieved in the transmission part of the network.

In addition, there has been a proposal for core slicing using NFV technology (Table 1). This involves the separation of network functions from specialized hardware. A network function (NF) serves as a functional component in a network infrastructure, possessing clearly defined external interfaces and exhibiting precisely specified functional behavior. Instead of the traditional approach, where NFs are tied to specialized hardware, these functions, after the mentioned separation, operate as virtual NFs on standard servers located in data centers [12]. This means that multiple network functions can run on a single server, utilizing the resources of a piece of hardware, as opposed to the traditional setup where each network function had its own dedicated hardware, consuming energy individually. In NFV, virtual network functions are connected through virtual links, which also have lower energy requirements compared to network functions on dedicated hardware with physical connections. From [9], it is evident that, by implementing NFV, energy savings can be achieved without the need for specific energy-saving algorithms or methods. As mentioned in [15], in the case of implementing the NFV concept, network energy consumption can be reduced by up to 50% compared to traditional telecommunications infrastructure.

When it comes to mobile networks, the highest energy consumption occurs in the RAN part of the network (Table 1). This accounts for approximately 70% of the total system energy consumption [1]. Therefore, different research efforts have aimed to improve the EE in the context of slicing the RAN part of the network. In [1], researchers propose a system for activating or deactivating RAN slices to establish a new slice with minimal activated resources and network elements, while maintaining a satisfactory QoS and low energy consumption. This approach can be realized in practice because slices are created for a specific type of traffic, service, or user, and can be deactivated when there is a significant reduction in the number of NS users and, consequently, NS traffic. However, the remaining active slice users need to be transitioned to the RAN elements of other active NSs. Deactivating the original slice increases overall network EE and the new slice to which users are migrated needs to use appropriate network resources to maintain the same or a slightly reduced QoS.

For the allocation of resources in network slicing (Table 1), researchers in [3] proposed two algorithms to achieve Pareto boundaries for two key network parameters: throughput and transmit power. These two algorithms are based on utility profiles and scalarization approaches, and the numeric outcomes of this paper illustrate the benefits of concurrently assigning bandwidth and transmission power, while also showcasing how QoS criteria tailored to individual segments influence both data transfer rates and the overall EE. This specifically means that these algorithms can provide optimal resource allocation in network slicing from the perspective of throughput and EE.

In [4], depending on the network services in NSs, strategies for managing EE to ensure its adaptability to the energy consumption patterns in 5G networks are presented (Table 1). This strategy suggests that network slices have priority identification that allows for normal service processing and the time-shift execution of services with a QoS degradation of some services or service rejection. The authors in [4] considered scenarios where energy is derived from renewable energy sources, meaning that a limited amount of energy is available, which makes it a limiting factor in service offerings. Therefore, the approach proposed in [4] cannot be applied to all types of slices, as some services are characterized by specific traffic activities, such as, for example, a service supporting autonomous vehicles. However, for slices enabling standard mobile services in 5G networks (such as voice, data, and video for commercial purposes, without excessive reliability requirements), the proposed strategies can be a significant step forward in terms of improving EE. In the case of a 5G mobile network realization without network slicing, the realization of an autonomous vehicle service and standard mobile services in standard non-sliced 5G network do not reduce energy consumption, leading to a less energy-efficient network.

The work addressing the impact of NS on E2E network EE is presented in [13] (Table 1). The optimization of throughput and resource allocation in the cloud was explored with the aim of minimizing the overall network energy consumption. The outcomes of the performed simulation demonstrate that comprehensive E2E network slicing is more energy-efficient than solely focusing on RAN slicing. According to their suggested methodology, there is the potential to achieve energy savings of up to 20% compared to relying solely on RAN slicing. Comparing the results from [12,13], a rough conclusion can be drawn that one-third of the energy consumption savings are achieved through core and transport network slicing, while two-thirds of the energy consumption savings are achieved through RAN slicing. This is in line with the results and data presented in [1].

Paper [14] introduces a model for the energy optimization of NSs using Integer Linear Programming (ILP). This model is designed to address the challenge of optimizing energy consumption while constructing an E2E NS in implementations with stringent security requirements (Table 1). Through simulation experiments, it was demonstrated that the proposed model achieves significant energy savings while meeting stringent QoS and security requirements. As the authors claim, the proposed model is suitable only for static NS environments, which diminishes the possibility of its practical implementation since real-world telecommunication networks are highly dynamic.

From the presented literature overview, it is evident that the use of network slicing allows for various strategies to save consumed energy and enhance the EE of 5G networks. Generally, significant differences in energy savings exist in NSs when E2E is applied comprehensively across all parts of the network. The presented research shows that there is an insufficient exploration of the impact of NS on the possible improvement in 5G networks’ EE. Therefore, the aim of this review work is to highlight the possibilities for improving the EE of 5G networks through the application of NS and to describe the current standardization of KPIs and other aspects related to enhancing the EE of 5G networks using NS as a concept.

## 3. Network Slicing in 5G Networks

### 3.1. 5G Network Slicing Architecture from the Perspective of Energy Efficiency

Network slicing is a concept in which multiple logical networks exist in one physical infrastructure in such a way that existing networks do not overlap while providing all functionalities as if they were established on separate physical networks. In the 3GPP specification [16], an NS is conceptualized as a logical network that is designed to offer specific capabilities and characteristics, accommodating a range of service properties tailored for customers utilizing NSs. According to [17], the concept of network slicing is based on different network resources that are sliced and allocated to each NS. Therefore, the NS is a specific set of network resources allocated to the UEs or use cases. The NS is thus a logical approach to allocating network resources along the data path into multiple network sets, which are optimized for specific use cases or groups of UEs. Network slicing allows different services to coexist on a common physical infrastructure. The dynamic nature of network slicing enables real-time adaptation to changing network conditions and service requirements. This adaptability is essential for providing reliable and high-quality services in dynamic environments. Each NS acts as an isolated entity, thus ensuring that the performance and security of one slice do not affect the others.

Every NS can be generally characterized by the following parameters:

1. Throughput: for the services specific to the NS, each slice should have a designated portion of throughput reserved for itself.
2. Topology: every NS is expected to possess a unique perspective on the network elements and the interconnections that bind these elements together.
3. Resources: every NS needs to have a guaranteed share of computational resources (processors, storage, communication links, physical networking elements, etc.) that can be used for NS service offerings.
4. Memory: since different NSs have different storage needs, memory resources need to be allocated according to the requirements of each NS.
5. Traffic forwarding: an NS needs to enable the extension of its service area, utilizing forwarding routing tables and other networking resources at the control layer.
6. Traffic type: specific traffic of the same type can be grouped and assigned to a single NS, which needs to ensure its complete isolation from the rest of the network and better overall resource utilization.

**Figure 3:**
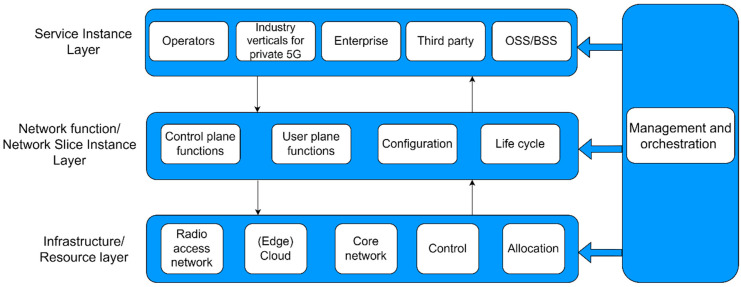
Concept of 5G network slicing [10].

The main concept behind network slicing, as shown in Figure 3, is built upon a framework of three distinct layers, as follows: the service instance layer, network function/slice instance layer, and infrastructure/resource layer [18].

**The service instance layer** is the layer that includes the services or virtual functions offered by the operators, enterprises, or third-party vendors that can be directly accessed by end users or businesses (Figure 3). Each distinct service is represented by a service instance that contains the operations system support (OSS) and business system support (BSS) elements of the service offered through NS. The service instance layer makes the lowest direct contribution to the overall NS energy consumption; however, the OSS/BSS politics and practices of the subject that offers specific services in NS can have an indirect impact on the NS instantaneous energy consumption and its future trends.

The **NS instance layer** is the layer that involves the instances of network slices that can be deployed (Figure 3), and includes elements related to the user and control plane functions, equipment configuration policy, and NS life cycle management. A network slice instance provides the required network features for the associated NS service instance’s functionality. The contribution of the NS instance layer to the overall energy consumption of NS is not negligible, since the configuration of the equipment, the amount of exchanged information for the support of user and control plain functions, and the lifecycle management of NS resources directly contribute to the overall energy efficiency of the NS.

**The resource layer** is the layer that provides all the essential virtual or physical resources and network functions needed to establish a service of NS instances (Figure 3) [19]. It includes all RAN, core, and cloud network elements, along with their corresponding communication links. The resource layer makes the highest direct contribution to the NS energy consumption and the main energy consumption optimization policies need to be implemented at this layer.

The **management and orchestration** (MANO) entity (in Figure 3) is responsible for translating service models and use cases into operational NS(s). The MANO handles dynamic management tasks, including the initiation, termination, and monitoring of an NS and its VNFs. This is necessary for achieving the real-time scaling of NS resources or auto-healing of potential issues during the operation of NS. The **management part of the MANO entity** involves predicting and regulating the usage of resources needed for NS functionality. It encompasses envisioning virtual resources, assigning resources to users including performance or diagnosing issues, and performing tasks related to solving these issues. The **orchestration part of the MANO entity** entails the anticipation and organization of the network resources needed to provide NS services. This includes assigning resources according to the requirements of applications or users, the distribution of workloads, dynamic adjustment of the network to accommodate traffic demands, and performing other comparable functions. Since the MANO entity spans all three layers of the NS architectural framework, it can serve as the main entity for monitoring and controlling the energy consumption of NS. In order to take part in all architectural layers of the NS, most implementation policies related to the optimization of NS energy efficiency can be executed through the MANO entity. Examples that implement NFV at different network levels in the current MANO platforms are ETSI NFV [20], while examples of the practical implementation of MANO controllers that enable the reconfiguration of resources to VNFs on the fly are the open network automation platform (ONAP) [21] or open-source MANO (OSM) [22].

### 3.2. Energy Demand of Network Slicing Service Types

Every NS in the 5G network is uniquely identified through the Single Network Slice Selection Assistance Information (S-NSSAI) identifier [23]. This identifier is signaled between the UE and the 5G network during the signaling procedure and it assists the network in selecting a particular NS instance that provides the UE with a specific service across the fixed/wireless access and core part of the network. The S-NSSAI consists of the eight-bit Slice/Service Type Identifier (SST ID) that specifies the network slice’s features and services and a 24-bit Slice Differentiator (SD), which is an optional element that, when combined with SST, provides additional information for distinguishing between multiple slices with the same SST. Currently, up to eight different signaling messages S-NSSAIs that are exchanged among the network and UEs are allowed by the 3GPP. This means a single UE may be served by a maximum of eight different NSs at the same time.

It is possible to use **non-standardized S-NSSAI**s that can use only SST ID alone or SST and SD identifiers, and this **non-standardized S-NSSAI** enables operators to specify their own services in specific NSs. However, to achieve global interoperability for NS, which enables MNOs to support roaming between NSs more efficiently, the SST values for the most commonly used NSs are standardized in [24,25,26,27]. Currently, standardized **S-NSSAIs** with only SST IDs are presented in Table 2, along with the general description of the main use cases of NSs and their energy demand. The currently standardized SSTs assume next NS use cases in 5G networks that include the following: enhanced mobile broadband connectivity (eMBB), ultra-reliable low-latency communication (URLLC), massive internet of things (MIoT), vehicle-to-everything (V2X) services, high-performance machine-type communications (HMTC), and high-data-rate and low-latency communications (HDLLC). In terms of the management of energy consumption, different SST types play an important role in the deployment and differentiation of mobile networks with NSs [4], since every NS SST ID or use case has a different energy demand due to differences in the practical implementation scenarios of a specific SST type. 

**The implementation of eMBB slices** is applicable to the existing mobile broadband networks and can expand the possible uses of the existing network towards a fully interconnected society. It is expected that emerging services, such as entertainment, gaming, virtual and augmented reality, video streaming, and fixed wireless access, will have a dominant representation in the 5G eMBB slice (Table 2). 

The **URLLC services** are related to the use cases characterized by a significant need for instantaneous interaction. For instance, public safety, remote medicine, emergency response, and smart grids are expected to be typical examples of NSs offering URLLC services (Table 2). However, network resources that need to ensure the functionality of eMBB and URLLC slices will have a high energy demand due to the large and frequent intensity of the data traffic that is characteristic for eMBB and URLLC slices. 

The **MIoT SST type represents massive machine-type communications (mMTC) slices.** This category encompasses cost-effective, energy-efficient, long-range machine-type communication (MTC) on the one side, and high-speed broadband MTC on the other side of the communication link. In the forthcoming years, lightweight and low-power sensors might be increasingly incorporated into everyday life, and consequently, this will lead to a notable increase in the deployment of sensing devices. Intelligent services will become ubiquitous in both urban and rural settings, facilitating tasks such as sensor networks, smart telemetry, smart homes, and the Internet of Everything (IoE) as typical expected examples of MIoT service. 

Additionally, **high-performance machine-type communication (HTMC)** is defined as another SST type, which includes low-latency, high-availability, and high-data-rate services without the need for any mobility or auxiliary links. Typical examples of HTMC NS are the implementations of industrial IoT, smart factories, smart cities, etc. It is expected that NSs that need to ensure the functionality of **mMTC** or **HTMC** will have a low energy demand due to the need for the sporadic transfer of data, during which the transmission policy can be adapted to reduce the NS resources’ energy consumption.

**Figure 4:**
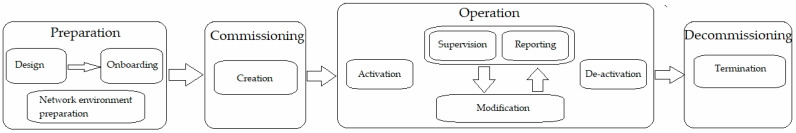
3GPP lifecycle management of a network slice [16].

In the realm of **Vehicle-to-Everything (V2X)** communication, vehicles are engaged in a bidirectional exchange with various Internet of Things (IoT) systems enabling autonomous driving, driver and pedestrian safety management, traffic management, road infrastructure management, etc. This shift is attributed to the progress in ICT and the swift evolution of IoT within the transportation sector. Within V2X communications, the data gleaned from IoT smart devices and other origins traverse through robust links characterized by low latency (within the sub-millisecond range [19]), high bandwidth, and reliability [28]. Additionally, in 3GPP Release 18, the introduction of the **high-data-rate and low-latency communications (HDLLC)** NS aims to provide a suitable response to the increased QoS demands of extended reality and multi-modality (XRM) streams. XRM services include concurrent video, audio, ambient-sensor and haptic data encryption, and data exchange in 5G networks. Network resources that need to ensure the functionality of the **V2X** and XRM slices will have high energy requirements, as shown in Figure 4. The 3GPP lifecycle management of a network slice’s is more demanding due to the need for high reliability and exploitability among the NS resources that are expected to be used in these slices [16].

### 3.3. Energy Efficiency in NS Lifecycle Management

The NSs implemented within operators’ networks must undergo comprehensive management throughout their entire lifecycle. According to [16], the 3GPP lifecycle management of an NS is composed of four phases: preparation, commissioning, operation, and decommissioning. Elements of these four phases are shown in Figure 4. From Figure 4, the **preparation phase** is the initial stage in the lifecycle of an NS and during this phase, there is no existing NS instance in the operator network. This stage of the NS lifecycle involves the design of the NS, capacity planning, onboarding, an evaluation of network functions, and organizing the network environment. Although not specified in the 3GPP NS lifecycle management specifications, NS energy consumption should be also considered as one of the factors in the NS planning phase. This is because energy-efficient NS planning can have a positive impact on the remaining phases of the NS lifecycle and can contribute to the reduction in NS energy consumption.

Furthermore, during the **commissioning phase**, the NS instance is created, and in this process, all necessary resources are configured and allocated with the aim of establishing the required capacities of the NS (Figure 4). In the **commissioning phase**, establishing the NS instance can involve both creating NS components and modifying them. To have as many energy-efficient NSs as possible, in the commissioning phase, the implementation of energy-efficient resource allocation techniques should take place.

The **operational phase** of an NS lifecycle encompasses NS commissioning, monitoring, generating feedback on performance quality and KPIs, utilizing adequate resources as needed, and modifying the NS instance according to users’ needs (Figure 4). Although the initial practical realizations of NSs energy consumption have not been considered as a relevant KPI, the energy consumption of NSs should be considered as an important KPI for the operation of the network and should be one of the KPIs that need to be monitored. This phase also includes deactivating and re-activating the NS when necessary. Activation puts the NS instance into operation for various communication services. This phase also engages the NS resources for supervision and reporting on the operation of the NS instance. Based on this supervision and reporting, modifications are made to the instance during NS operation. These modifications can involve changes to the capacity or topology of the NS, as well as the creation or modification of its components. Modifications may be initiated in response to new requirements for the NS or based on information obtained through monitoring and reporting. In this phase, deactivation involves temporarily terminating communication services and functions, representing the opposite action to the activation of the instance. From the perspective of network energy efficiency, the activation and deactivation of NSs according to the demand for network services or functions can contribute to the optimization of NS energy consumption. Therefore, significant network energy savings can be achieved during the operation phase of the NS lifecycle if appropriate operation approaches for optimizing NS energy consumption take place during the operational phase.

Finally, during the **decommissioning phase**, the provisioning of the instance involves deleting the configuration that was specifically created for this instance using the network element components (Figure 4). After this phase, the NSe instance ceases to exist [16]. From the perspective of the network energy footprint, the decommissioning phase should result in the full termination of resources that will not contribute to the future energy consumption of the network.

### 3.4. Energy Demand of NSs Provided as Services of Mobile Virtual Network Operators

**Figure 5:**
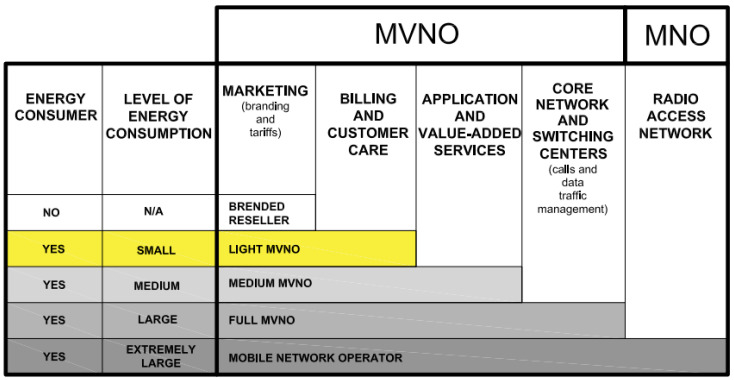
MVNO and MNO infrastructure and services [29].

In 5G mobile networks, a highly interesting use case of NS implementation is to offer NS as a service. By assigning a network slice as a service, organizations that do not own the infrastructure at all, or do not fully possess the mobile network infrastructure, can operate as mobile operators, and they are referred to as mobile virtual network operators (MVNOs). As illustrated in Figure 5, there are various versions of network slices used as a service, depending on the resources required by the MNO. They are generally defined as **branded reseller**, **light**, **medium**, and **full MVNOs**. However, the level of MNO resource engagement for the realization of MVNO services will impact the overall energy-consumption footprint of the NS that is realized as an MVNO. Consequently, the MNVO will have a different influence on the energy consumption policies of the corresponding NSs.

The **branded reseller** MVNO type is typically the most straightforward for an MNO to accept, as the MNO retains control over the majority of processes (Figure 5). In the case of a brand reseller, the MVNO NS lacks core network elements and is primarily responsible for maintaining contact and relationships with customers through service marketing and tariffing. The advantage of this approach is that it offers a quick service time to market and low startup costs to the MVNO, since there is no need for investment in the MVNO infrastructure as the MNO handles most of these aspects. The disadvantage of this approach to MVNO is the lack of control and subscriber identity modules (SIMs); additionally, the network infrastructure is owned by the MNO, which also sets tariffs for the services offered to the MVNO users. For the control and management of the network energy consumption policy, MNO takes full responsibility, and the energy costs are included in the pricing of the NS service for the MVNO. Thus, light MVNO is not a direct energy consumer, and all network energy consumption is related to the MNO.

A **light MVNO** operates using its own brand and is in charge of customer care and billing for the offered service(s), as well as having limited access to network routing (Figure 5). The MVNO introduces an NS service, contains a customer database, and promotes and distributes services which enable the MVNO to differentiate itself from competitors. One of the advantages of light MVNO in comparison with the brand reseller MVNO concept is that it owns branded SIM cards and can independently set tariff packages. However, the realization of the NS service offering incurs a share of the operational expenses (OPEX) and capital expenses (CAPEX) with the MNO. Despite knowing customers’ identities and SIMs, the MVNO does not possess the International Mobile Subscriber Identity (IMSI) of the users, which are owned by the MNO. The light MVNO has an impact on the control and management of the energy consumption of the small portion of those network elements it possesses, while the control and management of the energy consumption of most network elements leased from the MNO are in charge of the MNO. Thus, the NS, realized as the light MVNO, is an energy consumer; however, its energy consumption is significantly smaller than NS part of the network energy consumption of the whole MNO.

A **medium MVNO** operates as an NS under its own brand with its own proprietary SIM cards and has the potential to obtain its own mobile network code (Figure 5). While not fully independent from the MNO (e.g., MVNO interconnection and IMSI are controlled by the MNO), the medium MVNO has the ability to introduce its own services that enhance the sales of offered services. The advantage of medium MVNO compared to light MVNO lies in its independence from the MNO in creating and offering its own applications and value-added services to users, along with corresponding tariff packages and retail prices. This enables the MVNO to obtain a competitive market advantage by introducing applications and services not offered by competitors or the MNO. However, when compared to the light MVNO, the realization of medium MVNO incurs a significantly larger share of the OPEX and CAPEX. The medium MVNO has a nonnegligible impact on the control and management of NS energy consumption, since it possesses more network resources compared to light MVNO, while the control and management of the energy consumption of other network elements leased from the MNO are in the charge of the MNO. Thus, the NS realized as the medium MVNO is also an energy consumer, which energy consumption is smaller than the NS part of the network energy consumption of the MNO.

A **full MVNO** is realized as NS with a comprehensive standalone core network and switching elements that utilize the radio access network of other MNO(s) to connect user devices to its network (Figure 5). The full MVNO is a type of E2E NS where elements of the network, spanning from the core through the transport and up to the radio access part of the network, are sliced by the MNO that is to be leased by the MVNO. The main advantage of full MVNO is that it can incorporate its own network and application innovations into the leased NS, offering end-users services that the MNO does not possess. Moreover, this slice, with additional network and application advantages or innovations, can be offered again to another MVNO as a service. Additionally, the advantage of using a full MVNO as an NS is that it has all the functionalities of an MNO, while the drawback is its significantly high OPEX and its share in CAPEX. Compared to previously described MVNO types, another drawback is the necessity for extensive knowledge regarding the functioning and management of telecommunication systems of the full MVNO, which entails hiring skilled personnel. The stated significant share in the CAPEX of the full MVNO is also reflected in the full MVNO being a high energy consumer. Due to its ownership of more network resources than previously described MVNOs, the MVNO’s overall energy consumption is significantly larger compared to previously described MVNOs. However, the possibility of full MVNO to influence the energy consumption policy and management of network resources is also large.

## 4. Standardization of 5G Network Slicing Energy Efficiency KPIs

**Figure 6:**
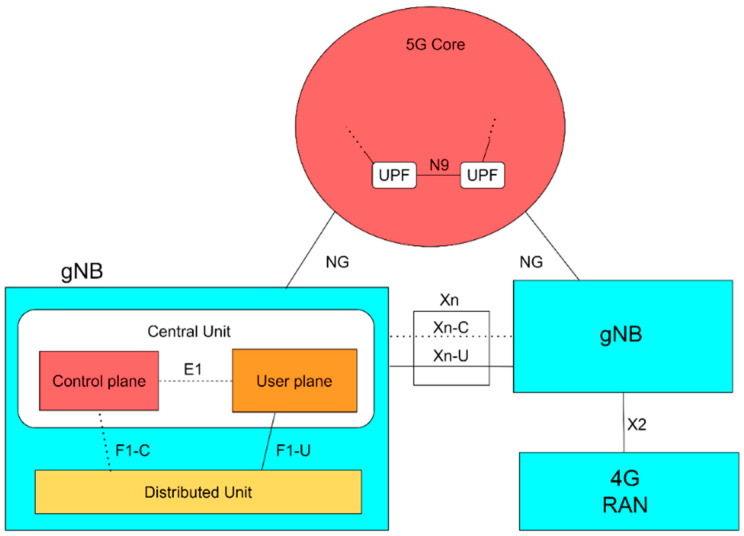
Standardized interfaces in the 5G network architecture [33].

Although the first standardization of 5G mobile networks occurred in 3GPP Release 15 [30], the KPIs related to the energy efficiency of 5G network slicing were initially addressed in 3GPP Release 17 [31]. While it is reasonable to assume that KPIs addressing the energy efficiency of 5G network slicing will be further extended in the ongoing 3GPP Release 18 and the upcoming 3GPP Release 19, the currently standardized KPIs in [31] provide a general definition of 5G NS energy efficiency. More specifically, standardized energy efficiency KPI for 5G networks is a metric that is expressed as the ratio of data volume (DV) traversing through NS and the energy consumption (EC) of a specific network part or the NS [32]. Data volume, used as a parameter for the calculation of 5G NS energy efficiency KPI, is measured on 5G network interfaces serving a specific NS. Figure 6 shows standardized interfaces in the 5G network architecture that are relevant for defining the energy efficiency KPIs of 5G network slicing.

The 5G network architecture is composed of a core network and base stations (BS), defined as next-generation Node B (gNB), in the 5G RAN part of the network (Figure 6). As shown in Figure 6, the NG is the physical interface between the 5G core network and gNBs, i.e., the RAN part. E1 is the logical interface between the control plane and the user plane in the central unit (baseband processing unit) of 5G gNB. Its function is to support the exchange of signaling information between the user and the control plane (endpoints). The F1-C interface defines the interconnection between the central unit (CU) and the distributed unit (DU) of the gNB. The DU is (an active) antenna element (AAE) or remote radio head (RRH) of gNB, which can belong to the same vendor or a different vendor than the CU. F1-U is the interface related to communication between the user plane of CU and the user plane of DU. The Xn is the interface through which communication occurs between gNBs. As in the previous case, the labels U and C in the case of this interface (Xn-U and Xn-C) refer to interfaces that enable communication between the user planes and control planes of gNB, respectively. The N9 interface is used to connect two UPFs in the core part of the network. The X2 interface connects the 4G RAN and the 5G RAN parts of the network. Also, in this interface, there is a control part and a user part, labeled as X2-C and X2-U, that are used for the transfer of control and user information between BSs in 5G and 4G networks, respectively.

In Table 3, the KPIs expressing the energy efficiency of various standardized NS SSTs are presented, with the description of key NS performance indicators and corresponding measurement units. As a metric of 5G network energy efficiency, the 3GPP standard [24] relies on the EE KPI defined in [34]. According to the standard, Mobile Network Data Energy Efficiency (${EE}_{MN,DV})$ is determined by calculating the ratio of the transferred data volume (*DV_MN_*) to the consumed energy (${EC}_{MN}$) of the network, with both metrics measured concurrently.

(1) $${EE}_{MN,DV}=\frac{{DV}_{MN}}{{EC}_{MN}}\;\;\;\;\;\;\;\;\;\;\;\;\;\;\;\;\;\;\;\;\;\;\;\;\;\;\;\;(bit/J)$$

Also, in [34], the Mobile Network Coverage Energy Efficiency (${EE}_{MN,CoA})$ is an additional KPI, defined as the ratio of the area covered by the observed mobile network (${CoA\_des}_{MN})$ and the consumed energy for the time period in which the efficiency assessment is performed.

(2) $${EE}_{MN,CoA}=\frac{{CoA\_des}_{MN}}{{EC}_{MN}}\;\;\;\;\;\;\;\;\;\;\;\;\;\;\;\;\;\;\;\;\;\;\;\;\;\;(m^{2}/J)$$

where ${EC}_{MN}$ is the annual consumed energy and ${CoA\_des}_{MN}$ represents the network coverage achieved by the analyzed network.

**Table 3:** KPIs for expressing the energy efficiency of standardized NS service/slice types [35].

KPI Name	Description of KPI Calculation	Performance Indicator	Unit (Remark)
Energy efficiency of eMBB network slice (SST type 1): *EE_eMBB,DV_*	This KPI is obtained by dividing the total amount of uplink (UL) and downlink (DL) data that pass through the N3 interface (Figure 6) of the NS by the consumed energy of the NS.	UL and DL data volume	bit/J
			(If there are redundant transmission paths across the N3 interface, it is anticipated that the data volume will be accounted only once.)
Energy efficiency of eMBB network slice—RAN-based (SST type 1): *EE_RANonlyeMBB,DV_*	A KPI indicating the energy efficiency of eMBB-type NS is derived from measurements of traffic. The *P_ns_* for an eMBB-type NS is calculated by dividing the aggregated uplink (UL) and downlink (DL) data volumes at the F1-U, Xn-U, and X2-U interface(s) of gNBs (Figure 6) on a per-S-NSSAI basis with the total consumed energy of the NS.	UL and DL data volume	bit/J
Energy efficiency of URLLC network slice (SST type 2): *EE_URLLC,Latency_*	This KPI quantifies the energy efficiency of the URLLC-type NS. *P_ns_* for URLLC represents the reciprocal value of the average end-to-end user plane latency for the slice. The primary focus of this KPI is latency, which serves as the sole criterion for assessing the NS’ performance.	Latency	(0.1 ms·J)^−1^
			(KPI is derived by taking the reciprocal of the average end-to-end user plane (UP) latency for the NS and dividing it by the energy consumption of the NS.)
Energy efficiency of URLLC network slice (SST type 2): *EE_URLLC,DV,Latency_*	*P_ns_* for this type of network slice is obtained by dividing the sum of UL and DL traffic volume on the N3 or N9 interfaces (Figure 6) by the value of the end-to-end user plane latency for that specific NS. This parameter is crucial for network operators when they want to assess EE in different time periods during which these two parameters vary.	Latency and data volume (DV)	bit/(0.1 ms·J)
			(If redundant transmission paths are implemented to enhance communication reliability, it is anticipated that the data volume will be accounted for only once.)
Energy efficiency of MIoT network slice (SST type 3): *EE_MIoT,RegSubs_*	*P_ns_* is determined by the maximum number of users who have registered in the NS. The calculation of this KPI is straightforward and represents the quotient of the maximum number of registered users to the total consumed energy of the NS.	Enrolled registered subscribers within the NS	user/J
Energy efficiency of MIoT network slice (SST type 3): *EE_MIoT,ActiveUEs_*	*P_ns_* is determined by the average number of users who are active in terms of utilizing the NS. The calculation of this KPI is realized by dividing the average number of active users (active UE) by the consumed energy of the NS.	Number of active users within the NS	User equipment per Joule (UE/J).

Clearly, ${EE}_{MN,CoA}$ can be applied only to the RAN part of the network, while the ${EE}_{MN,DV}$ metric is applicable across the different entireties (RAN, transport, or core) of the 5G network. These standardized KIPs are presented in relations (1) and (2) to express whether the energy efficiency of the 5G network can be directly implemented if only one E2E NS is realized in the 5G network. However, in most real implementation cases, NSs are created in the 5G network in such a way that more than one NS uses the common network element or communication link, and thus the 3GPP defines the adopted KPI to express the energy efficiency of NSs. According to the 3GPP standard [35], the KPI for expressing the energy efficiency of a generic network slice (${EE\;KPI}_{GNS})$ is defined as follows:

(3) $${EE\;KPI}_{GNS}=\frac{P_{ns}}{{EC}_{ns}}$$

In Equation (3), $P_{ns}$ represents the performance indicator of the NS, and its value depends on the key performance factor selected for analyses of that slice. For example, depending on the use case of NS, for some slices, the key factor can be throughput, while for other NSs it can be latency, etc. The unit of measurement varies depending on the slice, i.e., the key characteristics by which it is defined. Depending on the selected performance parameter, for example, for the eMBB NS (Table 2), the unit of measurement is bit/J, while the unit of measurement for the mIoT NS is user/J or user equipment/J.

In relation (3), the ${EC}_{ns}$ is the consumed energy of the NS. The 3GPP standard [35] defines NS energy consumption KPI, expressed in Joules (J), as follows:

(4) $${EC}_{ns}=\sum_{NF}{EC}_{NF}\;\;\;\;\;\;\;\;\;\;\;\;\;(J)$$

According to relation (4), the consumed energy of the NS is calculated as the sum of all energies consumed by each network function $\left({EC}_{NF}\right)$ executed in the NS. If this is an E2E NS, it is clear that it involves the network functions of entities from Figure 1 (except for UE) and that ${EC}_{ns}$ in (4) is equal to ${EC}_{ns}$ in (3). Additionally, the energy consumed by each network function ${EC}_{NF}$ of NS is calculated as the sum of all physical network functions and virtual network functions of NS and is expressed as follows:

(5) $${EC}_{NF}=\sum_{PNF}{EC}_{PNF\;measured}+\sum_{VNF}{EC}_{VNFestimated}\;\;\;\;\;\;\;\;\;\;\;\;\;\;\;\;\;\;\;\;\;\;\;(J)$$

The ${EC}_{PNF}$ represents the consumed energy on a physical element performing a specific network function that is not virtualized and is measured in accordance with [36]. ${EC}_{VNF}$ cannot be measured directly like ${EC}_{PNF}$ and the energy consumed by virtualized elements is estimated as described in [33,35].

Until the further standardization of new NS SSTs and corresponding energy efficiency KPIs, the parameters and models for calculating energy efficiency KPIs described in Table 3 can be used for other unstandardized NS implementations. The selection of the energy efficiency KPI calculation can be made based on the level of similarity between the implemented unstandardized NS and some of the standardized NS SSTs presented in Table 3.

## 5. Possibilities of Improving Energy Efficiency with Network Slicing

### 5.1. Operational States of NSs and Corresponding Resources

Apart from the necessity of establishing KPIs that facilitate the evaluation of the energy efficiency of the entire network or a specific NS, it is crucial for MNOs to manage the network resources supporting NS(s) to save energy while maintaining an appropriate QoS. However, in terms of network slicing, the 3GPP TS 23.501 [23] defines three terms: **NF, NS, and NS instance**. The **NF** is a processing function in a network that defines the functional behavior of an NS and which can be implemented either as a virtualized function installed on an appropriate platform (e.g., cloud infrastructure), as a software application executed on allocated hardware, or as a network element operating with the corresponding hardware. While an **NS** is a logical network that provides specific network characteristics and capabilities, an **NS instance** is a set of NFs and the corresponding resources (needed for, e.g., networking, storage, or computing) that form a deployed NS.

There are two operation states of NS resources or overall NS(s) standardized by 3GPP in [24], in which an RAN wireless cell, NS instance, NF, and an overall NS can be used. These are the energy-saving state and the operating state (which is a non-energy-saving state). Thus, the basic principle of any energy-saving mechanism in 5G network slicing is the transition during NS operation from a non-energy-saving state to an energy-saving state and vice versa. This transition must be accompanied by the principle that another resource element (NS, NF, RAN wireless cell, NS element, etc.) needs to accept the workload of those NS resource elements or the overall NS, which is in an energy-saving state. Furthermore, the energy-saving state of the NS resource element or the complete NS should not result in a QoS degradation and must prevent the overloading of the remaining active NS(s) or resource element(s) [24]. Considering the two previously mentioned energy-saving states, it is logical that there must be two fundamental procedures for entering and exiting these states. In the next sections, procedures related to improving the energy efficiency of 5G NS(s) will be explained in more detail.

### 5.2. Approaches to Improving Energy Efficiency of NSs in 5G Networks

The energy efficiency of a 5G NS can be improved by reducing the electricity consumption of 5G network resources dedicated to ensuring the functionality of NS(s). This reduction in electricity consumption also results in OPEX reductions in MNOs. Such an improvement in the energy efficiency of NS(s) in 5G networks can generally be achieved in three ways, which are categorized as the reengineering of **network equipment, the dynamic allocation of network resources, and smart sleeping** [37]. These three approaches can be applied in 5G network slicing separately or combined to a greater or lesser extent.

This work will not analyze **reengineering,** as this is beyond the scope of this paper since it involves the use of energy-efficient materials and components for developing more energy-efficient equipment hardware and the reduction in device complexity, which tends to ensure that the same performance can be achieved with fewer electronic elements and thus a reduced energy consumption. However, the **dynamic adaptation of network resource allocation and smart sleeping,** as prominent approaches to improving the energy efficiency of 5G NSs, will be analyzed in detail in the remaining sections of this work. **The dynamic allocation of network resources** involves the scaling of NS(s) resources in such a way that NS(s) resources that are not necessary for service delivery will not be utilized. In the **smart sleeping** approach, specific NS elements are completely shut down to achieve energy savings and they are restarted as needed [38,39]. Also, key enablers of network slicing include SDN, VNF, and cloud computing. The SDN separates the control plane from the physical network elements, which results in a reduced energy consumption by the physical network elements. An overview of the research topic presented in Section 2 shows that centralizing NS control in one location allows for more efficient control compared to each NS element having its own control plane. This leads to reduced energy consumption for each NS element, which has a positive impact on the overall energy consumption of the entire NS.

In addition, it is shown that the implementation of VNF technology in network slicing also has a positive impact on NS energy efficiency. This impact is reflected in the ability to implement multiple NFs on a single physical device through the virtualization of network resources, and thus achieve equivalent effects to the realization of NFs on separate physical devices. In this regard, the implementation of VNFs requires virtual links among NS resource elements, which have a lower energy footprint compared to physical links. In addition, cloud computing is another key enabler of network slicing, which can ensure even more energy-efficient NS resource utilization. In cloud computing, the more efficient use of processors, storage, and cooling systems reduces heat generation or overheating, which has a positive impact on NS energy efficiency. It is worth mentioning that data centers performing cloud computing often rely on renewable energy sources, ultimately reducing costs for MNOs and contributing to a reduction in energy consumption from non-renewable sources.

#### 5.2.1. Resource Allocation in 5G Network Slicing

**Figure 7:**
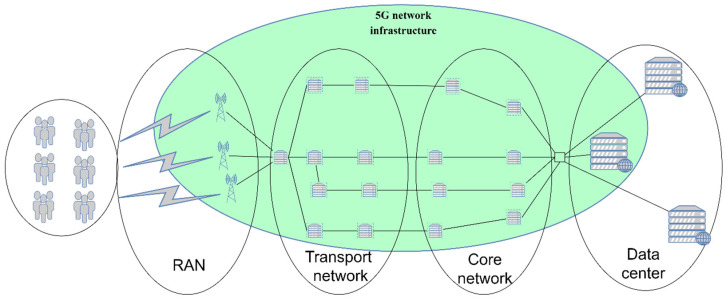
Non-sliced 5G mobile network segments.

In the case of legacy 5G networks realized without network slicing (presented in Figure 7), some users may demand all types of traffic, ranging from those with high QoS demands to those with low QoS demands. In such a configuration, the entire 5G network uses significantly more resources than necessary, since the network must be dimensioned according to the users demanding the highest QoS parameters. This leads to an increase in network energy consumption, while the amount of traffic passing through the network remains largely unchanged and a large amount of network resources actively operate at low utilization levels. Although individual network elements (NEs) in the 5G networks are generally more energy-efficient than the network elements of previous network generations (better power consumption per unit of transferred traffic), the densification and capacity increases in the installed 5G network elements result in an increase in the overall energy consumption of the 5G network when compared to networks of previous generations and this increase raises the OPEX of MNOs. For example, one macro 5G BS, although ensuring significantly larger capacities, requires nearly 70% more energy than a macro BS that, in the same rack, combines second-generation (2G), third-generation (3G), and 4G technology [40].

Although the possibilities of improving 5G networks’ energy efficiency are limited, the use of network slicing in 5G networks can contribute to this improvement. One of the main approaches to improving 5G NS energy efficiency is 5G network resource allocation through the implementation of the 5G network slicing concept with the help of orchestrators, which are an integral part of the MANO entity (depicted in Figure 3). NSs can be created and orchestrated based on **soft or hard network slicing**. In soft slicing, NS can share some network resources with other NSs, while in hard slicing, NSs must be fully isolated from each other. Thus, two fundamental mechanisms used for adapting network resources are slice isolation and orchestration. For example, with hard slice isolation, slice B cannot use resources assigned to slice A, thus preventing an increase in energy consumption for slice A. Slice B may use the same physical network element where a network function is enabled for slice A, but how much energy each VNF on that physical element consumes for slice A and slice B must be precisely known. Slice orchestration can thus dynamically add to or reduce the resources of the NS(s) in a smart manner or an artificial intelligence (AI)-driven manner.

By grouping users with similar traffic requirements and allocating appropriate resources only to specific types of traffic or service, along with the use of the mechanisms of slice isolation and orchestration, and by putting certain network elements into sleep mode through resource reallocation, network slicing can influence the dynamics of the usage of network elements and their corresponding capacities. As stated in the previous section, energy efficiency KPIs are defined as the ratio of network slice performance and consumed energy. Therefore, to increase these KPIs, increasing the NS performance and/or reducing energy consumption are possible ways to improve NS energy efficiency. The dynamic adaptation of network resources and resource reallocation primarily influence the increase or decrease in NS performance, while the smart sleep mode affects the reduction in consumed energy.

**Figure 8:**
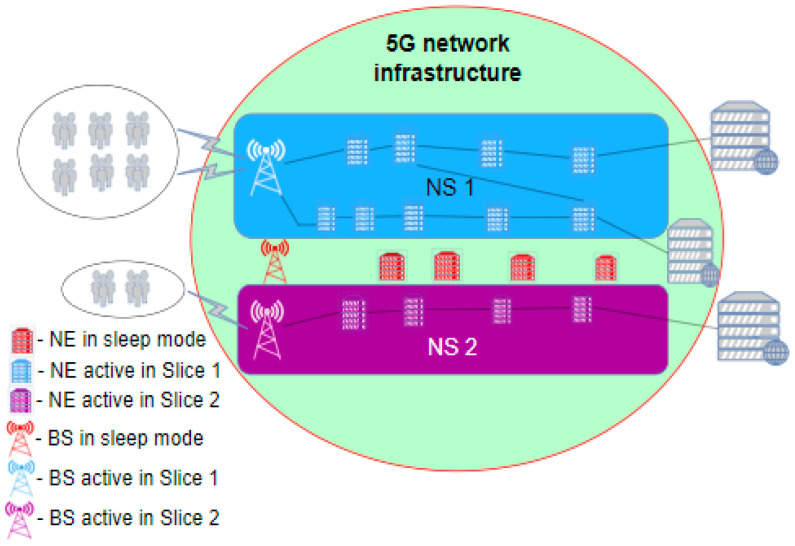
Allocation of network elements (NEs) for two NSs in the 5G mobile network.

The stated approaches to improving 5G NS energy efficiency are shown in the example illustrated in Figure 8, which represents two NS in a 5G network. Users are grouped in NSs according to the type of communication service they use. The network configuration of these slices is such that the required QoS of the services offered to the users must be provided. As can be seen in Figure 8, during the establishment of NSs, it is possible that some network elements may not be used at all, given that both slices satisfy the specified QoS parameters with the allocated network resources. Thus, these non-used resources of the 5G network are not essential for the services offered by NSs. As these elements can enter sleep mode while working, energy consumption in the entire 5G system will be reduced in comparison with the unsliced legacy 5G network presented in Figure 7. Thus, through NSs’ orchestration (NSs’ management), the adaptation of the 5G network resources to the offered service in the NS is achieved by adding or reducing necessary resources to preserve the required QoS while ensuring an improvement in network energy efficiency.

**Figure 9:**
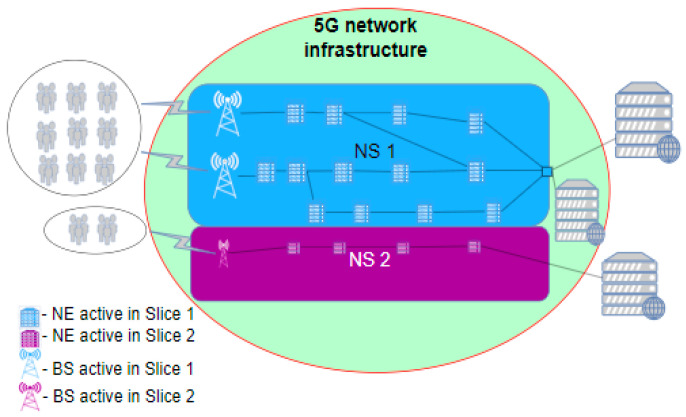
New network elements (NEs) allocation in the 5G network for NS1.

If there is an increase in the traffic of some NS, for example, in Slice 1 (Figure 8), additional resources are engaged through MANO at that moment to handle all traffic demands, as presented in Figure 9. Since a much higher volume of traffic is processed in the NS, the performance of the slice is raised to a significantly higher level. However, this additional engagement of network resources will lead to an increase in consumed energy due to the additionally activated network elements. If we consider the NS energy efficiency KPI, it is clear that the network performance, compared with the scenario presented in Figure 8, has increased in the scenario presented in Figure 9 to a level of performance equal to the one presented in Figure 7, and consequently the energy consumption also increases to the same levels. This leads to the conclusion that the adaptation of network resources in network slicing can contribute to an increase in energy efficiency, since in the periods of 5G network activity presented in Figure 8, the network consumes less energy when compared with the legacy 5G network presented in Figure 7 or the sliced network presented in Figure 9.

**Figure 10:**
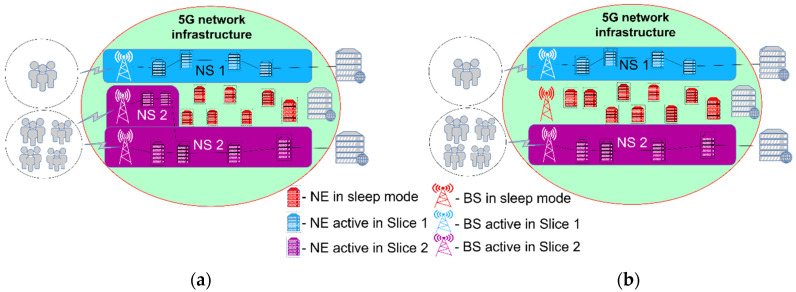
Allocation of network elements (NEs) in the 5G network with two network slices for the case of: (**a**) traffic increase in NS 2; (**b**) traffic decrease in NS 2.

Additionally, in a situation where there is a reduction in traffic or service demand in some NS(s) compared to the configuration with fully activated NS(s) resources presented in Figure 9, the adaptation mechanisms of the MANO entity can act to reduce the use of 5G network resources. In this situation, the resources freed up by adaptation mechanisms can be in two states, either being used by another NS due to increased traffic demands in that NS, as presented in Figure 10, or put into a sleep state, as presented in Figure 10b. In both cases, the energy efficiency of the 5G network will be improved in comparison with the efficiency of the legacy unsliced 5G network presented in Figure 7, since at least some elements of the 5G network resources will be in the sleep operation state (Figure 10) and thus in an energy-saving state.

#### 5.2.2. Network Slices Realized as Network Subnets

**Figure 11:**
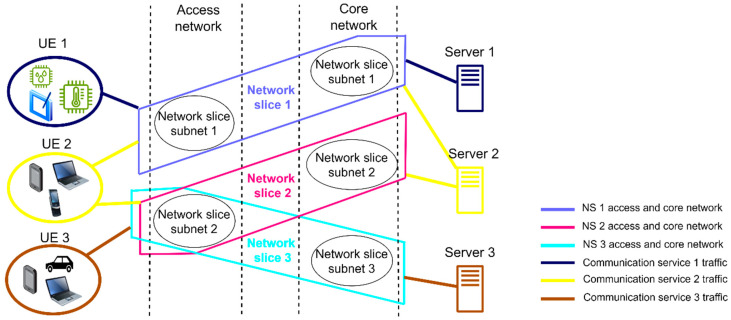
Multiple communication services realized through multiple network slices [16].

In the previous examples, hard network slicing is presented, where the NS resources are entirely used in the communication service taking place between the UE and the VNF of the server, which serves one NS in the access, transport, and core network elements, and thus NS resources cannot be shared among different NSs. Therefore, each NS is tied to the specific service that is offered to the users through the exploitation of network resources solely by this NS. This approach to creating network slicing is one of the possible approaches to setting the 5G sliced network. In addition, 3GPP TS 28.530 [16] states that different NSs can use the common subnet in the access or core part of the 5G network. Figure 11 illustrates the core and access network parts of the 5G network that constitute specific NSs for illustrative purposes. In this case, different parts of the NS subnets can be used in different parts of the 5G network (access or core).

In the case of the example in Figure 11, there are three NSs (NS1, NS2, and NS3) with three communication services on Servers 1, 2, and 3, which are offered to the three different groups of users—UE1, UE2, and UE3, respectively. The services are realized through NS1, NS2, and NS3, and their corresponding subnets span over different parts of the access and core network (Figure 11). The 3GPP standardization presents the possibility of more than one NS using one subnet in the access or core part of the network at the same time (e.g., NS2 and NS3 use the same subnet in the 5G access network, as illustrated in Figure 11). This example of soft network slicing opens further opportunities for reducing energy consumption in the 5G network. Two or more NSs sharing the same network resources enables better 5G network resource exploitation and opens the possibility of putting more 5G network elements in the sleep (energy-saving) operation mode.

An interesting case of using such soft network slicing to achieve possible improvements in the 5G networks’ energy efficiency appears in the example, presented in Figure 11, where a communication service offered by Server 2 to the user’s group UE2 utilizes the core and access network resources of two NSs (NS1 and NS2). In the case of a traffic scenario where a low traffic volume of NS2 is transferred over the 5G network during a certain period(s) of the day, there is potential for energy savings to be achieved by putting NS2 core network subnet elements into sleep mode, while the communication service of NS2 between the UE2 and VNFs of Server 2 can be transferred over the core network subnet of NS1. In this case, the NS1 core and access network subnet need to adapt their capacities to ensure an appropriate QoS for NS2 and its own NS1. This opens a new field of research dedicated to the development of algorithms for the dynamic activation and deactivation of parts of the core or transport/access networks that serve specific NS according to variations in the traffic intensity of specific NS services. In the next section, some of the possible approaches to improving 5G network energy efficiency through the dynamic adaptation of network resources in network slicing are presented.

### 5.3. Limiting Network Slice Energy Consumption

**Figure 12:**
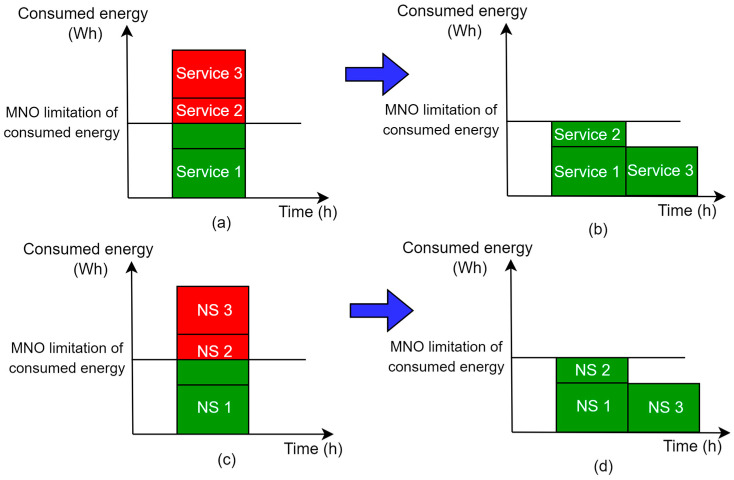
NSs energy consumption approaches based on: (**a**) no limitation on maximal energy consumption per NS service; (**b**) limitation on the energy consumption of NS services and scheduled energy consumption imposed by MNO; (**c**) no limitation on maximal energy consumption per NS; (**d**) limitation on NS energy consumption and scheduled energy consumption imposed by MNO.

One of the options for improving the energy efficiency of the 5G sliced network is an approach dedicated to limiting and reallocating the energy consumption of specific NS service(s) or complete NSs. The MNO can limit the maximum consumed energy for a particular NS or NS service(s). Figure 12a,c illustrate an example of an MNO energy consumption policy without energy consumption limitations for three services of one NS or three NSs, respectively.

However, in case of limitations on overall NS(s) energy consumption being defined by the MNO, services can be realized through the degradation of the QoS to a minimally acceptable level for some services or NSs (Figure 12b,d). This energy-saving approach based on the degradation of QoS can be implemented in those services or NSs that do not demand high reliability, or have low traffic latency and can postpone the processing of data. Also, the QoS can be degraded for some services during a short period of time. However, if the degraded QoS persists, the QoS can become unacceptable and the maximum energy consumption threshold for specific service(s) or NS(s) should be adjusted by enabling the activation of additional network resources which will maintain an acceptable level of QoS. This leads to the necessity of developing techniques for the dynamic adaptation of the maximal energy consumption threshold for specific services or NSs according to the need to accommodate specific QoS standards of service or NS.

In addition to service degradation and dynamic maximal energy consumption adaptation principles, it is possible to apply an algorithm for the temporal shifting of service execution or NS resource reallocation outside the time period of maximal energy consumption and complete execution of degraded service. Figure 12b,d illustrate a situation where limitations on the service or NS energy consumption were applied to service 2 or NS2, respectively, for the amount that exceeded the energy limitation. Also, Figure 12b,d present an example of a scenario where service 3 and NS3 had their execution and resources’ reallocation temporally shifted in the periods outside of the maximal energy consumption threshold. This temporal shifting of the service execution approach can be implemented in practice, since users may be aware of the shifted service execution for specific services. This approach in turn can provide lower service costs to the users, as the MNO reduces expenses regarding energy consumption. Thus, the development of innovative algorithms for the temporal shifting of service execution or NS resource reallocation outside the time period of maximal energy consumption is also a possible way to optimize 5G networks’ energy consumption.

On the other hand, in practical implementations of NSs and corresponding services, there are services that should be prioritized in terms of execution. This can be achieved by the MNO, which will determine service or NS execution priorities, thereby deciding which of them takes precedence in terms of the usage of resources of the 5G network. However, prioritization of the execution of some services or the operation of NSs themselves may cause new limitations in terms of a lack of available energy in some parts of the 5G system, due to the increased energy consumption of resources needed for the realization of prioritized NS services or NSs. In that case, priorities can be set to determine which service or NS has an advantage in terms of energy consumption, and consequently in the usage of network resources. Other, lower-priority services or NSs may experience temporal degradation or a temporal shifting of service execution due to the inability to engage sufficient 5G network resources. However, such cases will require immediate action to reduce negative impacts on QoS in specific parts of the 5G system. Therefore, this energy-consumption balancing for each service of NS or for the overall NS(s) will require new approaches and techniques that need to be investigated with the goal of achieving the optimal energy consumption distribution among resources of NSs.

### 5.4. Improving Energy Efficiency in the RAN Part of 5G Network Slices

Considering that almost 70% of the 5G network energy is consumed in the RAN part of the network or, more specifically, by the BSs, the main focus of research on reducing the energy consumption of 5G networks should be on techniques that enable the energy-efficient allocation of RAN resources among NSs. According to [41], BS resources remain unused for 75–90% of their operation time, even though they can occasionally experience significant traffic loads. The field of improving the energy efficiency of the RAN part of different network generations (2G, 3G, 4G, and 5G) has been extensively analyzed in the literature [2,38,42,43]. Thus, many technologies and techniques proposed or used for improving the energy efficiency of the RAN part of the 5G network can be adjusted for application in sliced 5G networks.

In 5G sliced networks, the RAN part of the network can be dedicated to a specific NS or can be shared among different NSs. This means that BSs, as the major energy consumers in the RAN part of the network, can serve only one NS or can be shared among several different NSs. Although use cases of BSs serving multiple NSs will be dominant in practice, one NS being served by one BS is particularly interesting in the case of implementations of micro, pico, or femto BSs in private 5G networks acting as a standalone NS. With the introduction of the 5G new radio (NR) standard, complete BS or different BS components can enter sleep mode to achieve energy savings. The possibility of optimizing NSs energy consumption in the RAN part of the 5G network depends on the level of sleep state that BSs or specific components can achieve. the general sleep state depths of BSs or BS components are categorized as macro- and micro-sleep states and different BS components are more suitable for different sleep state depths (Table 4). The duration of micro-sleep states is in the order of microseconds or milliseconds, while the durations of macro-sleep mode are longer and can span up to hours or even days. A more practical means of achieving macro-sleep mode is using BS elements such as a remote radio head/unit (RRH/U), active antenna element (AAE), power amplifier, baseband unit (BBU), or complete BSs (Table 4). The BS components that are more suitable for the micro-sleep mode are carrier frequency blocks, subcarrier frequencies, wireless channels, and orthogonal frequency division multiplexing (OFDM) symbols. 

In general, the energy-saving process based on putting the complete BS or a BS component serving one or more NSs into sleep mode depends on three aspects: different triggers and scenarios for entering or exiting sleep mode, the duration of the sleep mode, and the depth of the sleep state. Thus, the optimization of NS energy consumption in 5G networks will require algorithms for adjusting BS parameters supporting NS(s) operation. Such algorithms need to enable BS or its component to frequently enter or exit sleep mode, while the duration of the sleep mode is impacted by the data and signaling traffic intensity of the NS served by a specific BS component or the whole BS. The depth of the sleep state (spanning from macro- to micro-sleep) is achieved by using different amounts of BSs components not serving any NS into sleep state. This can range from partial shutdown to the complete shutdown of certain BS components, with the possibility of restarting the BS components when necessary. Restating BS components from sleep state, however, takes longer if the depth of the sleep state is greater [44]. Thus, the trigger for the BS or BS component serving specific NSs to enter/exit the sleep state, and the depth and duration of the BS or BS component’s sleep state (to achieve the required energy savings of the 5G network), can be directly influenced by the character of the NS service(s), its traffic and signaling intensity, and QoS constraints. In addition, energy savings in the RAN part of the 5G network can be achieved in the time, space, and frequency domains. The possibilities for energy savings in the sliced 5G RAN in each of these domains are analyzed in further sections.

#### 5.4.1. Possibilities for Energy Savings in Sliced 5G RAN in the Time Domain

In the time domain, possible energy savings are at the level of OFDM symbol shutdown. In the case of BSs serving a single NS, the activity of power amplifiers (PAs) when there is no transmission of OFDM data symbols can be significantly reduced, as well as the frequency of transmitting control signals.

**Figure 13:**
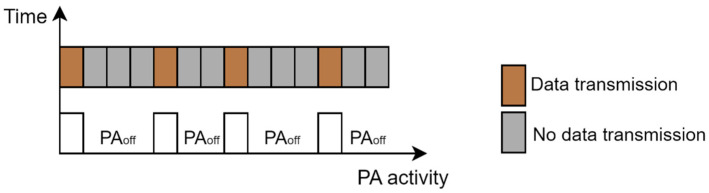
The activity of the BS power amplifier according to data transmission variations [44].

As shown in Figure 13, a basic principle of energy saving is shutting down PA during periods with no data OFDM symbol transmission. This concept has already been implemented for new generations of 5G BSs in unsliced 5G networks, and it is shown that such a concept can contribute to the reduction in BS energy consumption. It is worth emphasizing that this type of energy saving can be implemented when the BS is moderately loaded, which means that switching the PA on and off can only be allowed in the periods when a lower amount of traffic is transferred over the NS. In the case of intensive data traffic being transferred in NS, every symbol’s time slot will be occupied, and this reduces the possibility of shutting down PAs [45].

**Figure 14:**
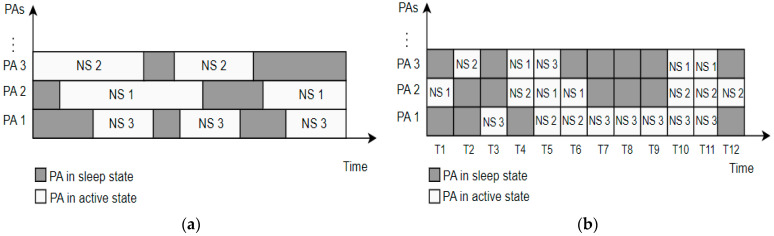
Allocation of BS PAs to specific NSs: (**a**) permanently; (**b**) based on the scheduling of PAs among NSs.

Additionally, in the 5G sliced network with BSs simultaneously serving multiple NSs, to obtain further energy savings, the concept of scheduling PAs’ activity can be extended to the distribution of PAs among different NSs, as presented in Figure 14.

One concept is based on an approach where a specific PA is permanently allocated to specific NSs, as presented in Figure 14a, while another approach is based on the time scheduling of NSs traffic transfer among different PAs (Figure 14b). In both concepts, energy savings are obtained for specific NSs in periods when there is no data traffic for transfer in the NS. The implementation of such a concept will require the development of techniques that will enable the dynamic scheduling of PAs among NSs on the same BS or will require the possibility of allocating the specific PA to the specific NSs through BS configuration.

#### 5.4.2. Possibilities for Energy Savings of Sliced 5G RAN in the Spatial Domain

Spatial energy savings in 5G sliced networks can be obtained through the implementation of the Multiple-Input–Multiple-Output (MIMO) technique. MIMO, as a technique, is based on wireless transmission over arrays of multiple antennas, which results in reduced signal fading, improved signal-to-noise ratio at the wireless communication ends, and the ability to create multiple communication streams that boost channel capacity over a finite electromagnetic spectrum. The implementation of multi-user MIMO (MU-MIMO) and massive MIMO (mMIMO) enable the further enhancement of channel capacities. The densification of antenna arrays in the MIMO system enables the implementation of energy-saving techniques through the spatial allocation of wireless streams over arrays of MIMO antenna elements.

**Figure 15:**
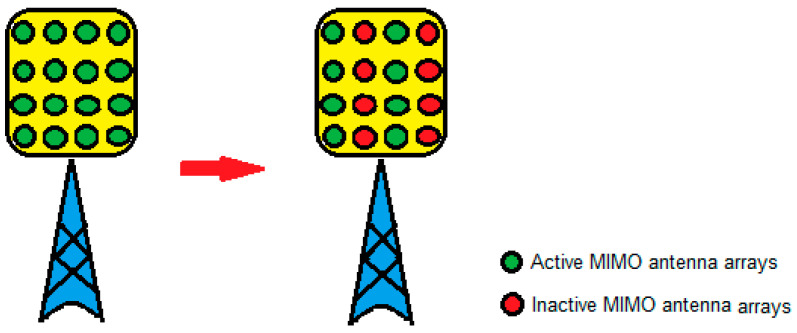
MIMO spatial energy saving based on the scheduling of antenna array activity states [44].

The basic idea of creating energy savings in the spatial domain is related to the switching on and off of MIMO antenna arrays according to traffic intensity. During time periods when the activity of all MIMO antenna transceivers is not necessary as there is low or no traffic intensity, a certain number of MIMO antenna arrays can be turned off (Figure 15) if switching off antenna elements will not affect the overall user experience.

Considering that 5G generally uses more antenna arrays, especially when utilizing MU-MIMO and mMIMO, possible energy savings in the spatial domain can be significant. After turning off certain transceivers representing channel transmitters, there will be a reduction in the antenna total transmission power and antenna gain. This can be compensated by increasing the spectral power density of the remaining active transceivers of the MIMO antenna array [45].

**Figure 16:**
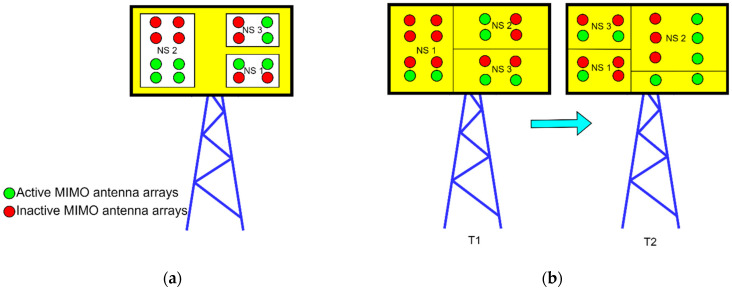
Allocation of BS antenna array elements to specific NSs: (**a**) permanently; (**b**) based on the scheduling of antenna array elements to be shared among NSs.

To enable further energy savings through the implementation of the 5G sliced network, there is the possibility of allocating different MIMO antenna arrays to the NSs. The possible configuration of the MIMO transmission in the 5G sliced network can be seen in Figure 16.

It can be noted that two approaches dedicated to the allocation of NSs to MIMO antenna arrays can be implemented in practice. The first one is based on a dedicated configuration of a specific set of MIMO antenna arrays for specific NSs (Figure 16a), while the second approach encompasses the possibility of the dynamic scheduling of different MIMO antenna arrays among NSs (Figure 16b). Every option includes turning off some MIMO antenna array elements that are allocated to specific NS, in order to reduce the energy required by a specific NS (Figure 16).

The practical implementation of the dynamic allocation of NSs among different antenna array elements with the ability to switch off some antenna array elements to save energy will require new approaches and algorithms that can support such implementations. However, the use of massive MIMO antennas in 5G sliced networks according to the proposed approaches can lead to a reduction in energy consumption. The conventional use of antennas in mobile networks has been characterized by signal emissions in specific sectors covering a vast area. This results in a significant percentage of the emitted signal energy being unused. With the proposed approach, based on the scheduling of NSs among massive MIMO antenna elements, electromagnetic wave emissions are directed to the users of specific NSs, and there is no energy wastage as occurs with conventional MIMO transmission with sector antennas. When combined with the techniques that support turning off some MIMO antenna array elements during periods of low traffic activity, network slicing can provide significant energy consumption savings to 5G networks.

#### 5.4.3. Possibilities for Energy Savings of Sliced 5G RAN in the Frequency Domain

Possibilities for improving the energy efficiency of 5G sliced RAN also exist in the frequency domain. Two approaches are seen as possibly being able to increase the energy savings of 5G networks in the frequency domain. The first one is based on completely turning off the 5G signal carrier, while the second one is based on reducing the bandwidth of the carrier by turning off some subcarriers. Considering the extension of the 5G operating frequency spectrum up to 6 GHz and the possibility of using the millimeter wave spectrum, the concept based on scheduling radiofrequency spectrum resources to be shared among NSs in the frequency domain to improve 5G energy efficiency gains new significance.

This is particularly evident in areas where there is an overlap between the signals of 4G and 5G networks [44]. In situations where traffic in the 5G network is very low, users can be transferred to the 4G network by turning off the 5G carrier through the X2 interface (Figure 6). Once control mechanisms detect an increase in these users’ activity or an increase in traffic demands, the carrier in the 5G network is reestablished through control mechanisms to accommodate these users and their traffic requirements [45].

**Figure 17:**
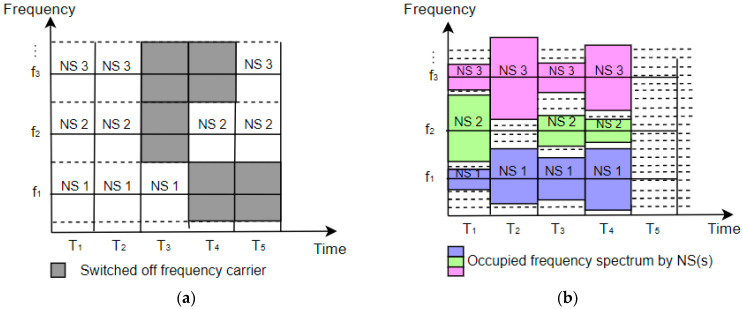
Allocation of the frequency spectrum to specific NSs: (**a**) permanently; (**b**) based on the scheduling of frequency spectrum resources to be shared among NSs.

The presented approach, which is already implemented in new generations of 5G BSs, can be extended to 5G network slicing to improve 5G networks’ energy efficiency. In Figure 17, two approaches to the allocation of the frequency spectrum to specific NSs are presented. In Figure 17a, the carrier frequencies and available spectrum (bandwidths) are statically allocated through the VNF configuration of the BS to the specific NS. In the case when there is no data traffic in specific NSs, the frequency carrier of that NS can be switched off and thus operate in energy-saving mode. In Figure 17b, the dynamic spectrum (bandwidth) allocation of specific frequency carriers to a corresponding NS is performed in accordance with the overall frequency spectrum availability and current bandwidth needs of the NS. In traffic situations when an NS requires a lower bandwidth, the allocation of a reduced channel bandwidth through the reduction in used subcarriers can contribute to the energy savings of the BSs serving that NS(s).

### 5.5. General Artificial Intelligence-Based Process for Optimizing the Energy Efficiency of 5G Sliced RAN

**Figure 18:**
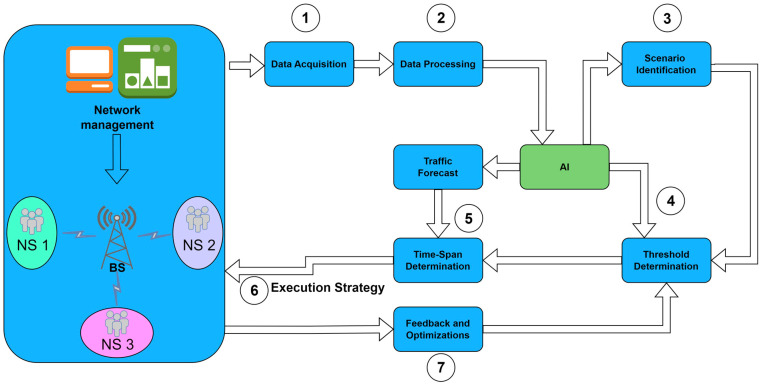
AI procedures for energy consumption reductions in RAN serving NS(s) [45].

The possible strategies for energy savings in 5G sliced networks in the time, spatial, and frequency domains presented in the previous sections can be realized only with advanced management and orchestration strategies for 5G RAN. To achieve such strategies, the use of artificial intelligence (AI) emerges as a potential approach for reducing the energy consumption of 5G NSs in the RAN. An overview of the general procedure of intelligent energy-saving strategies based on AI in the RAN network is presented in Figure 18 [45]. The AI-based procedure for improving the energy efficiency of 5G NS(s) in the RAN network is composed of several phases. The first phase is dedicated to obtaining relevant data about the performance of the BSs in an RAN serving NSs through control mechanisms (Figure 18).

After data collection, the data are processed through filtering and adapted to a format that is applicable for further processing. Such data are fed to the AI software, which creates the AI algorithm(s) and generates an operation scheme (scenario) for a specific NS, with the aim of optimizing the network energy consumption. Based on the goals that need to be achieved, in this phase, energy-saving KPIs and thresholds are also set (Figure 18). Following this, in phase 5, using historical data, the AI predicts the type and amount of traffic that will be present in a specific period of NS operation, and determines the energy-saving technique(s) and the timeframe for their application (Figure 18). In step 6, the chosen energy-saving strategy, which can be time-, space-, or frequency-based (or a combination of these), for a specific NS is sent to the control system as VNFs and executed utilizing the SDN technique (Figure 18). After implementing the selected strategy, in the final phase, phase 7, the performance of NSs is monitored to assess whether the energy efficiency goals set for a specific NS have been achieved (Figure 18). This AI-based procedure for energy consumption reductions in RAN needs to be implemented in a closed-loop system, which includes continuous adjustments of the energy efficiency KPIs and thresholds for a specific NS (as indicated in step 4).

From the presented AI procedure (Figure 18), it can be seen that specific RAN resources of the entire 5G system are allocated to each NS. The management of these resources, with the aim of optimizing energy consumption, is handled in the same manner through the exploitation of AI-based algorithms. It is important to emphasize that the AI-based procedures for energy consumption reductions can be applied separately to the RAN subnet of each NS. Also, if more NSs served by the same BS are comprised at the same time with the energy-saving scheme selected by the AI algorithm, the entire BS can be in the energy-saving mode of operation.

#### Resource Provisioning in Network Slicing Based on Artificial Intelligence

The main challenge in the practical realization of the described AI-based procedure for improving network energy efficiency (Figure 18) is the accomplishment of phases 5 and 6. Phase 5 is related to the prediction of the type and amount of traffic that will be present in the NS for a specific time period, while in phase 6, the resources that will offer appropriate energy savings have to be selected. These traffic prediction and energy-saving techniques are directly related to the NS resource allocation that needs to be provisioned to ensure an appropriate QoS and, consequently, impacts the NS energy consumption.

In [46], the authors study two migration policies of virtual router selection in sliced networks, defined as local traffic prediction based on only local knowledge of the traffic profiles of a specific network segment, and global traffic prediction based on the knowledge of the complete daily traffic profile of an operator. It has been shown that global traffic prediction achieved better results, by 35%, in terms of energy consumption reductions and revenue loss due to QoS degradation in comparison to local traffic prediction.

The problem of the appropriate selection of network resources is reflected in the possible over-provisioning costs due to reserving more resources (network elements, network functions, virtual machines, etc.) than needed. Conversely, under-provisioning costs are reflected in the increased network operator expenses for the degradation of QoS. At present, matching resources to VNFs is mostly a reactive process that uses hysteresis thresholds to ensure the efficient utilization of network resources. Thus, the usage of AI in the allocation of network resources among NSs is still in its early stages. Some preliminary results related to the usage of AI for optimizing resource allocation among NSs are shown in paper [47], where the authors propose an AI-based asymmetric Long Short-Term Memory (LSTM) resource allocation algorithm. The cost function of the proposed algorithm incorporates the cost penalty and takes into account network resource allocation costs and QoS degradation. It was shown in [47] that the proposed prediction algorithm in the NFV scenario with four network function virtual interfaces led to cost improvements of 40%. Another AI-based, proactive, and automated data analytic tool that optimizes network resource utilization among NSs is proposed in [48]. An AI-based machine learning solution has been developed that exploits space- and time-independent correlations of mobile traffic and provides MNOs with information about the capacity needed to satisfy the demands of every network slice. The proposed solution computes outputs at a data center level through a customized loss function that predicts capacity needs, and thus enables MNO to reduce the possibility of resource overprovisioning or underprovisioning.

Nevertheless, the usage of AI in the allocation of network resources among NSs to improve network energy efficiency is still in its infancy. There is a need for additional investigations that will result in the development of new AI-based algorithms and techniques that will enable the integration of AI in the allocation of network resources among NSs, with the aim of optimizing network energy consumption.

### 5.6. Spatial Arrangement of Network Elements for Enabling Energy-Efficient Network Slicing in 5G RAN

**Figure 19:**
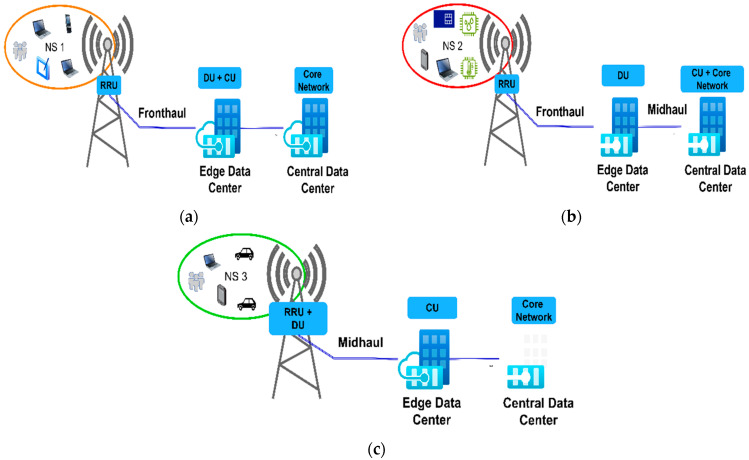
Architectures of 5G RAN and core elements for (**a**) NS with eMBB service type, (**b**) NS with mMTC service type, and (**c**) NS with V2X service type.

The latest 5G RAN architectures are characterized by separating the baseband unit (BBU) and remote radio head/unit (RRH/U) as two main elements of the 5G BS. This means that BBU and RRU (RRH) can be implemented in different physical locations (Figure 19). Furthermore, the 5G new radio introduces split BBU architecture, where the BBU functionality is generally divided into the functions of the DU and CU. In practice, the DU and CU do not have to be implemented at the same physical location (Figure 19). This means that the BBU functionalities of DU and CU can be distributed or implemented in the cloud. While the link known as the fronthaul enables an optical connection between DU and the RRU (RRH), the link known as midhaul is a packet-based optical fiber link that connects the CU and DU in the 5G RAN architecture (Figure 19).

With the advent of VNF and SDN as the main enablers of 5G network slicing, the DU and CU, as BBU functionalities, can be shared among different NSs in the 5G sliced network (Figure 19). This can enable the precise allocation of DU and CU resources to specific NSs. Figure 19 illustrates some possible combinations for implementing the RRU (RRH), DU, and CU in the RAN and core network for a 5G BS serving a specific NS. With respect to the energy efficiency of the 5G sliced network, the arrangement of these network components will depend on the type of traffic that will be transmitted through that network. In sliced 5G networks, the types of traffic are generally known in advance, as the traffic of a specific NS is grouped in this NS and transmitted through the respective 5G network resources. For example, for eMBB, mMTC, and V2X service types (Table 2), the recommended 5G network slicing architectures are the ones presented in Figure 19a, Figure 19b, and Figure 19c, respectively.

Since the DU and CU in the new BBU architectures presented in Figure 19 can be physically separated from the RRU/H (BS), they can be implemented in the edge or central data centers (DCs). This implementation of BBU functionalities in DCs allows for the virtualization of the DU and CU functionalities per specific NSs on DC virtual machines or containers. This usage of precise virtual DC resources for a specific BBU functionality and the possibility of quickly scheduling these resources to be used by different NSs, opens different possibilities for improving the energy efficiency of 5G BSs BBUs in comparison with legacy BBU realizations based on the fixed allocation of BBU on the BS site. Also, the implementations of DU and CU in the DCs can benefit from the DC-implemented mechanisms and techniques for optimizing energy efficiency. These can even include a power supply of DC from a renewable energy source. Moreover, the modern cooling methods of DC equipment are mostly in use for the entire DC, which reduces the costs of using separate cooling devices for the air conditioning of individual BBUs at specific BS sites that are characteristic of legacy 5G and previous network generations. These possible energy savings are furthered by the realization of BBU elements as VNFs, which enables the faster processing of necessary functions and for BBU elements to be put into a sleep mode of operation when they are not processing data and signalization.

The functions of CU and DU in the architecture of the split BBU RAN model presented in Figure 19 are defined by the division of OSI layers. This enables flexible use case performance optimization, scalability, and load management. For example, as presented in Figure 19b, by shifting non-real-time functions that are processor-intensive to more CUs that are centrally located, the routing of NS traffic and RAN resource utilization can be made more efficient. On the other hand, by shifting DU and/or CU functions closer to the RRU/RRH for use cases with high bandwidth demands and fixed user locations, such as those of eMBB service types (presented in Figure 19a), or for use cases with very low latency, such as V2X use cases (presented in Figure 19c), a significant improvement in information processing efficiency can be achieved. Compared with 5G nonsliced BS architectures, this efficiency improvement will also be reflected in the more optimal energy consumption of RAN resources serving NSs.

Although Figure 19a–c present use cases with one NS having a specific BBU architecture that is from the energy-efficiency point of view more suitable for the realization of specific SST, the concept of virtualization of DU and CU can enable having different NSs on the same location (RRU, edge or central DC) with different DU, CU and core network functions. For example, in the edge DC, one NS can have DU and CU (Figure 19b) at the same time, while the other NS can have only CU at the edge DC, with the DU located in the core DC (Figure 19b), or can have only CU at the edge DC, while the DU is near the RRU/RRH (Figure 19c). Therefore, for the improvement in 5G RAN energy efficiency through network slicing, the BBU components (DU and CU) need to be allocated according to the service characteristics of the NS (SST). Therefore, an appropriate allocation of BBU elements can contribute to energy efficiency improvements in 5G sliced networks.

## 6. Conclusions

The paper provides and overview of different aspects of 5G network slicing to improve 5G networks’ energy efficiency. Different types of 5G network slice use cases, defined as 5G service set types, have been presented and analyzed in terms of the energy consumption of specific use cases. Also, KPIs standardized as the metrics for 5G NSs’ energy efficiency are defined and elaborated for each 5G network slice use case. The paper further sheds light on ideas for future research on topics related to improving the energy efficiency of 5G networks utilizing network slicing. A plethora of options for the development of new techniques and algorithms that could impact the reduction in the energy consumption of 5G networks through the implementation of the network slicing concept are proposed. The discussed options include techniques such as limiting network slice energy consumption by mobile network operators and implementing dynamic changes in the operational states of NSs and corresponding resources according to variations in the traffic in an NS. Additionally, comprehensive analyses dedicated to possibilities for improving energy efficiency in the RAN part of 5G network slices through the implementation of methods for achieving energy savings by exploiting network resource adaptation techniques in the time, frequency, and space domains have been presented. The paper also overviews the AI-based processes’ ability to optimize 5G sliced RAN energy efficiency. Case studies exploiting the use of AI-based techniques in network slicing to improve mobile network energy efficiency are discussed. Lastly, the impact of the spatial arrangement of baseband unit elements in the cloud data centers for the improvement of 5G network energy efficiency through the implementation of 5G RAN network slicing has been elaborated.

It is shown that each of the techniques exploiting network slicing analyzed in the paper can contribute to the improvement in 5G network energy efficiency. Therefore, the improvement in 5G network energy efficiency through the application of one or a combination of the analyzed techniques based on network slicing represents an area with significant research potential, which can contribute to the further energy consumption optimization of energy-demanding 5G networks. This improvement is particularly important as energy is mostly derived from non-renewable sources. We ultimately aim to not only reduce the operational costs of mobile network operators, but also to reduce their negative impact on the environment.

## Figures and Tables

**Table 1:** An overview of research areas for improving energy efficiency through the implementation of 5G network slicing.

Reference	Technology/Approach Enabling Network Slicing	Research Possibilities for EE Improvement
[11]	SDN-based network slicing	Traffic-aware, end-host-aware, and rule-placement approaches using SDN scheduling
[12]	VNF-based network slicing	Virtual network functions’ scheduling
[1,3]	RAN-based network slicing	RAN resource scheduling
[4]	Network slicing based on the traffic’s adaptation to energy consumption patterns	Time-shift service execution of NS with possible QoS degradation and the power supply of NS derived from renewable energy sources
[13,14]	Ent-to-end (E2E) network slicing with stringent security requirements	NS scheduling through transport, the core, and the radio part of the network with guaranteed security requirements

**Table 2:** Standardized SST ID values for different use case examples and estimated energy demands [23].

SST ID Type	SST ID Value	Characteristic		Use Case		NS Energy Demand/Capacity/
eMBB	1	Enhanced Mobile Broadband Connectivity (eMBB) slice optimized for managing 5G enhanced mobile broadband services		Entertainment, gaming, virtual and augmented reality, video streaming, fixed wireless access		High
URLLC	2	Slice designed for ensuring ultra-reliable low-latency communication (URLLC) (e.g., 1 ms)		Public safety, remote medicine, emergency response, smart grid		High
MIoT	3	Slice tailored for managing extensive (Massive) Internet of Things (MIoT) applications		Sensor networks, smart telemetry, smart homes, Internet of Everything (IoE)		Low
V2X	4	Slice crafted for handling Vehicle-to-Everything (V2X) services		Autonomous driving, driver and pedestrian safety management, traffic management, road infrastructure management		Very high
HMTC	5	Slice suitable for facilitating high-performance machine-type communications (HMTC)		Industrial IoT, smart factories, smart cities		Low
HDLLC	6	Slice engineered for managing high-data-rate and low-latency communications (HDLLC)		Extended reality and multi-modality services (video, audio, ambient-sensor and haptic data)		Very high
**SST ID** **Type**	**Area Traffic Capacity**	**Peak/** **Experienced** **Data Rate**	**Spectrum** **Efficiency**	**Mobility**	**Latency**	**Connection Density**
eMBB	High	High	High	High	Medium	Medium
URLLC	Low	Low	Low	High	High	Low
MIoT	Low	Low	Low	Low	Low	High
V2X	High	Medium	Medium	High	Low	High
HMTC	High	High	Medium	Medium	Medium	High
HDLLC	High	High	Medium	Low	Low	Medium

**Table 4:** Categorization of 5G BS components as candidates for operation in sleep mode.

Macro-Sleep Mode	Micro-Sleep Mode
Remote radio head/unit (RRH/U)	Frequency blocks
Active antenna element (AAE)	Subcarriers
Power amplifier (PA)	Wireless channels
Baseband unit (BBU)	OFDM symbols
Complete BS	

## Abbreviations

Abbreviation	Definitions
2G	Second Generation
3G	Third Generation
3GPP	Third-Generation Partnership Project
4G	Fourth Generation
5G	Fifth Generation
6G	Sixth Generation
AF	Application Function
AI	Artificial Intelligence
AAE	Active Antenna Elemant
AMF	Access and Mobility Function
AUSF	Authentication Server Function
BBU	Base Band Unit
BS	Base Station
BSS	Business System Support
CAPEX	Capital Expenses
CN	Core Network
CO2	Carbon Dioxide
CU	Central Unit
DC	Dana Center
DL	Down Link
DU	Distributed Unit
DV	Data Volume
E2E	End to End
EC	Energy Consumption
EE	Energy Efficiency
EEMNDV	Mobile Network Data Energy Efficiency
eMBB	enhanced Mobile Broadband Connectivity
ES	Energy Saving
gNB	Next-Generation Node B
HDLLC	High-Data-rate and Low-Latency Communications
HMTC	High-performance Machine-Type Communications
ICT	Information and Communication Technology
ILP	Integer Linear Programming
IoT	Internet of Things
IMSI	International Mobile Subscriber Identity
KPI	Key Performance Indicator
LTE	Long-Term Evolution
MANO	Management and Orchestration
MIMO	Multiple-Input–Multiple-Output
MIoT	Massive Internet of Things
mMIMO	Massive MIMO
MU-MIMO	Multi-User MIMO
MTC	Machine-Type Communications
mMTC	massive Machine-Type Communications
MNO	Mobile Network Operator
MVNO	Mobile Virtual Network Operators
NE	Network Element
NEF	Network Exposure Function
NFV	Network Function Virtualization
NG-RAN	Next-Generation Radio Access Network
NR	New Radio
NRF	NR Repository Function
NF	Network Function
NS	Network Slice
NSA	NonStand Alone
NSSF	Network Slicing Selection Function
OPEX	Operational Expenses
OFDM	Orthogonal Frequency Division Multiplexing
OSS	Operations System Support
PA	Power Amplifier
PCF	Policy Control Function
QoS	Quality of Service
RAN	Radio Access Network
RRH	Remote Radio Head
RRH/U	Remote Radio Head/Unit
RRU	Remote Radio Unit
SA	StandAlone
SD	Slice Differentiator
SDN	Software Define Network
SIM	Subscriber Identity Modul
SMF	Session Management Function
S-NSSAI	Single-Network Slice Selection Assistance Information
SST ID	Slice/Service Type Identifier
TCAM	Ternary Content Addressable Memory
TN	Transport Network
UDM	Unified Data Management
UDR	Unified Data Repository
UE	User Equipment
UL	Up Link
UPF	User Plane Function
URLLC	Ultra-Reliable Low-Latency Communication
V2X	Vehicle-to-Everything
VNF	Virtual Network Function
XRM	Extended Reality and Multi-modality
